# Gonadal Development and Its Influencing Factors in the Red Swamp Crayfish (*Procambarus clarkii*): A Review

**DOI:** 10.3390/biology14091138

**Published:** 2025-08-27

**Authors:** Yuchi Wang, Dengge Xu, Chao Guo, Tanglin Zhang, Jiashou Liu, Wei Li

**Affiliations:** 1 State Key Laboratory of Breeding Biotechnology and Sustainable Aquaculture, Institute of Hydrobiology, Chinese Academy of Sciences, Wuhan 430072, China; wangyuchi@ihb.ac.cn (Y.W.); xudengge@ihb.ac.cn (D.X.); guochao@ihb.ac.cn (C.G.); tlzhang@ihb.ac.cn (T.Z.); jsliu@ihb.ac.cn (J.L.); 2 College of Advanced Agricultural Sciences, University of Chinese Academy of Sciences, Beijing 100049, China; 3 Hubei Engineering and Technology Research Center of Paddy Field Integrated Cultivation, Wuhan 430072, China

**Keywords:** *Procambarus clarkii*, gonadal development, environment, nutrition, and hormones factor, intensive breeding

## Abstract

The red swamp crayfish (*Procambarus clarkii*) is a crucial freshwater aquaculture species in China, and the reproductive capacity and farming efficiency are critically dependent on gonadal development. Consequently, the acceleration and synchronization of gonadal maturation constitutes fundamental prerequisites for intensive seedling breeding. This review systematically synthesizes current knowledge on gonadal development and its regulatory factors in *P. clarkii*, specifically addressing (1) histological characteristics, (2) developmental stages and cyclical patterns, and (3) key genes involved in gonadal regulation. We particularly focus on the combined effects of intrinsic and extrinsic factors—including temperature, photoperiod, nutrition, and endocrine regulation—on gonadal maturation. This comprehensive assessment offers both theoretical insights into gonadal development mechanisms and practical guidance for enhancing intensive seedling production in this economically vital species.

## Simple Summary

The slow advancement of intensive seed breeding techniques for red swamp crayfish *Procambarus clarkii* is a critical bottleneck for the sustainable development of China’s industry. Achieving accelerated and synchronized gonadal maturation is paramount for intensive seedling production in *P. clarkii*. This review consolidates the current understanding of gonadal development and its regulation in *P. clarkii*, focusing on the histological characteristics, developmental stages and cycles, and key regulatory genes. Particular attention is paid to the combined influence of intrinsic (e.g., hormones) and extrinsic factors (e.g., temperature, light, and nutrition) on maturation. Significant knowledge gaps persist, especially concerning male gonadal development mechanisms and the molecular basis of regulation. To bridge these gaps, prioritized development of synchronized maturation techniques and accelerated protocols is essential.

## 1. Introduction

The red swamp crayfish (*Procambarus clarkii*), a decapod crustacean of the family Cambaridae (Arthropoda: Crustacea: Decapoda), has become one of China’s most economically important freshwater aquaculture species. Originally endemic to the Gulf Coast region of North America (United States–Mexico border), the species was first introduced to Nanjing, China, via Japan in 1929 [1] and has since become naturalized throughout the country’s freshwater ecosystems. Recent decades have witnessed exponential growth in *P. clarkii* aquaculture, driven by strong market demand and its adaptability to various farming systems. China’s Ministry of Agriculture and Rural Affairs (2024) reported that *P. clarkii* ranked fourth in most productive freshwater aquaculture species nationwide, with an annual production of 3.16 million tons in 2023. This remarkable production volume underscores its crucial role in China’s aquaculture industry and rural economy [2].

Currently, China’s *P. clarkii* aquaculture industry is mainly dependent on the integrated rice–crayfish culture [3], with crayfish seedlings originating predominantly from the self-propagation and self-breeding of populations in integrated rice–crayfish culture systems and pond culture systems [4]. This production paradigm faces three fundamental constraints: (1) lack of standardized artificial breeding protocols, (2) unpredictable reproductive output and quality, and (3) seasonal limitations on seed availability. The large-scale intensive artificial breeding technology for crayfish seedlings is still underdeveloped. Intensive artificial breeding of *P. clarkii* encompasses a sequence of interdependent technical stages, including the cultivation of high-quality broodstock, selection of mature broodstock, mating and spawning, controlled fertilization and egg incubation, and larval rearing and juvenile management. Each stage critically influences the overall breeding success. While high-quality mature broodstock provide the essential foundation for effective reproduction [4], meticulous management of the environmental parameters (e.g., water quality, temperature) during incubation and optimal larval nutrition during rearing are equally vital for achieving a high survival and growth rate [5]. Gonadal development is a core process in the growth and reproduction of *P. clarkii*, essential for forming high-quality mature broodstock. Research indicates that the peak period for gonadal maturation and mating of *P. clarkii* in the middle and lower reaches of the Yangtze River in China occurs from August to September [6]. However, in natural environments, the variability in gonadal development and asynchronous maturation often leads to prolonged mating periods, high broodstock mortality, and inconsistent spawning. These factors, namely asynchronous gonadal maturation and high mortality, significantly compromise the seedling quality and efficiency of intensive breeding [7]. The gonadal development of *P. clarkii* is regulated by various factors, such as endocrine hormones and environmental conditions, which interact to significantly influence this process. Therefore, understanding the mechanisms and influencing factors of gonadal development in *P. clarkii* is crucial for advancing biological research and the intensive production of seedlings. This article systematically reviews the gonadal development of *P. clarkii* and its influencing factors, aiming to deepen the understanding of its developmental patterns and underlying mechanisms. It also explores future research directions to provide a theoretical basis for the efficient and intensive breeding of *P. clarkii* seedlings.

## 2. Gonadal Development in *Procambarus clarkii*

### 2.1. Testis Development in Procambarus clarkii

#### 2.1.1. Histological Study of the Testis

The testis of *P. clarkii* serves as the site for spermatogenesis [8]. Located within the cephalothorax near the dorsal region, the testes are positioned above the hepatopancreas and below the pericardial cavity, adjacent to it. The testes form a Y-shaped single structure with three distinct lobes: two anterior and one posterior, connected by elongated collecting ducts at their posterior ends, forming a unified structure with a milky-white coloration. The morphology of *P. clarkii* testes is commonly described as “Y”-shaped [9]. This shape contrasts distinctly with the slender annular testes of *Macrobrachium nipponense* [10] and the elongated rod-like testes of *Cherax quadricarinatus* [11]. Such morphological differences reflect the distinct biological traits among these species.

The testes of *P. clarkii* primarily consist of seminiferous tubules and collecting ducts [12]. Seminiferous tubules form spherical units (10–16 μm diameter) housing asynchronously developing germ cells, enveloped by connective tissue. Collecting ducts comprise a three-tiered system: primary ducts connect to individual tubules, secondary ducts, where 5–8 primaries converge, and tertiary ducts, which form a central collecting lumen. Functional asymmetry exists in the vas deferens: the right vas deferens maintains a patent connection to tertiary ducts, with secretory epithelium (anterior/middle) and a thickened muscular wall (posterior) for sperm transport and spermatophore formation; the left vas deferens is obstructed by epithelial filaments at the duct junction, rendering it non-functional for sperm transfer (Table 1) [9].

#### 2.1.2. Morphology of Procambarus clarkii Sperm

The spermatozoon of *P. clarkii* is a non-flagellated non-motile cell [13], whose ultrastructure exhibits significant taxonomic characteristics, comprising an anterior acrosome and a posterior nucleus. Radial arms envelop the acrosome, and the entire structure is encased in an extracellular capsule. Notably, compared to *Astacus* spp. (family Astacidae) and *Cherax* spp. (family Parastacidae), the *P. clarkii* acrosome (family Cambaridae) possesses a distinct acrosome spike. This spike is potentially involved in gamete recognition and mediates passive adhesion to the egg [13,14].

#### 2.1.3. Sperm and Testes Development Stages

Spermatogenesis in *P. clarkii* can be distinctly classified into six sequential stages (Table 2). Histological analysis demonstrates that the testis comprises numerous seminiferous tubules. The testicular development exhibits significant annual cyclical variations, and based on the predominant type of germ cells within seminiferous tubules during specific periods, the annual testicular developmental cycle can be clearly divided into five stages (Table 3). This categorization explicitly delineates the spermatogenic process and maturation cycles [9,15].

### 2.2. Ovarian Development in Procambarus clarkii

#### 2.2.1. Ovarian Histology

The ovary of *P. clarkii* is situated dorsally along the midline of the cephalothorax, closely appressed to the intestine above and the pericardial cavity below, while being enveloped laterally by the hepatopancreas. Together, these structures occupy the entirety of the cephalothoracic region. Unlike the bilobed ovaries typical of most shrimp species [16,17], the ovary of *P. clarkii* is trilobed, exhibiting a distinctive “Y”-shaped morphology [18].

The ovary of *P. clarkii* primarily consists of the ovarian wall and oocytes. The ovarian wall, serving as the outer membrane, is composed mainly of loose connective tissue and contains abundant blood vessels and sinusoids, which facilitate the transport of nutrients and oxygen. This structural feature is consistent with that observed in *Litopenaeus vannamei* [19]. The ovarian wall gradually thins as the ovary matures and thickens again after ovulation. The oocytes are enclosed by the inner membrane and germinal epithelium, the latter of which differentiates and proliferates oogonia. The number of oocytes is higher during the early stages of ovarian development but decreases as the oogonia grow, only to increase again post-ovulation. Initially, the ovary is small, with tiny tightly packed oocytes. As development progresses, the ovary expands, and the oocytes enlarge and become more separable. A pair of oviducts extends from each side of the ovary, running along the outer wall of the pericardial cavity and terminating at the base of the third pair of thoracic legs, forming the female gonopore. The diameter of the oviducts increases with gonadal development [20], and the secretory content within the oviducts shifts from basophilic to acidophilic. King [21] proposed that the oviduct secretes a lubricating fluid that facilitates egg release. However, the precise mechanism and process by which these secretions promote ovulation remain unclear, warranting further investigation.

#### 2.2.2. Ovarian Development Patterns

Most studies indicate that *P. clarkii* spawns once annually, with peak spawning periods typically occurring between September and October and occasionally from April to May [7,15]. Jin et al. [22] and Oluoch [23] observed that populations in Hubei Province, China, and Louisiana, USA, primarily spawn from August to November, peaking in October, while those in Lake Naivasha, Kenya, exhibit year-round spawning [23].

The ovary of *P. clarkii* undergoes distinct color changes as it develops. Initially transparent, the ovary transitions through stages of grayish-white, pale yellow, yellow, brown, and dark brown, eventually becoming deep reddish-brown [18]. Research indicates that ovarian coloration is primarily driven by carotenoid accumulation, notably astaxanthin deposition. This process concomitantly alters the physiological status, including enhanced antioxidant capacity and elevated hemocyanin levels, which may collectively contribute to the pigmentation mechanism [24]. The shift from transparency to bright yellow may also correlate with the synthesis of vitellogenin in the ovary [25]. Additionally, the formation of carotenoid–protein complexes can indirectly influence ovarian color changes in crustaceans [26]. In summary, the color transformation reflects the coordinated regulation of various substances and serves as a reliable indicator of vitellogenesis and ovarian maturation. Furthermore, studies have demonstrated that *P. clarkii* exhibits significant sexual dimorphism in energy allocation for gonadal development: females accumulate substantially higher lipid reserves, predominantly as triglycerides, during spring compared to males. This disparity is attributed to the higher energetic investment by females in subsequent vitellogenesis and ovarian maturation [27].

#### 2.2.3. Stages of Oocyte and Ovarian Development

Currently, the division of the oogenesis period in shrimp is mainly based on the size and shape of oocytes and whether yolk synthesis has begun in the oocytes [21,28], which is generally considered to be four phases (Table 4).

**Figure 1:**
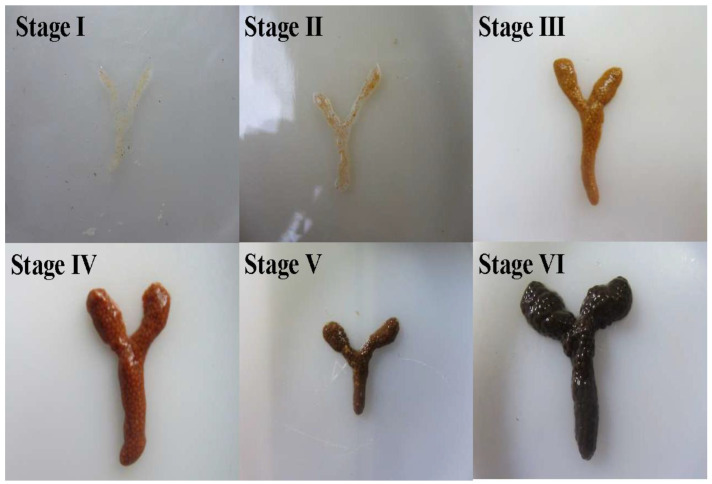
Ovarian development stages of *P. clarkii*.

The staging of ovarian development in shrimp is primarily based on histological and morphological characteristics [29,30], such as the external appearance, size, and coloration [31,32]. As shown in Figure 1, according to this staging method, the ovarian development of female *P. clarkii* is typically divided into six stages (Table 5).

### 2.3. Genes Associated with Gonadal Development in Procambarus clarkii

Potential genes influencing crustacean gonadal development include those involved in vitellogenesis (e.g., *Vg*, *VgR*), meiosis (e.g., *DMC*, *Cyclin B*, *CDC2*), sex differentiation and maintenance (e.g., *FOXL2*, *Dmrt1*, *Sox9*), and cell proliferation (e.g., *Pcna*). Extrinsic factors such as environmental temperature and nutritional status precisely modulate endogenous hormone levels, particularly neuro-endocrine hormones, via the neuro-endocrine system, rhythmically up- or down-regulating the transcription of these genes and thereby jointly orchestrating gonadal ontogeny and maturation [36].

Vitellogenin (VTG), a high-molecular-weight glycoprotein, is converted into vitellin (Vn) to provide energy for ovarian development. The vitellogenin gene (*Vg*) is closely associated with ovarian development [37]. Mardhiyyah et al. [38] determined that *Vg* is widely expressed in tissues such as the hepatopancreas of *Macrobrachium rosenbergii*, and its synthesis is closely linked to ovarian maturation. Similarly, Li et al. [39] demonstrated that the expression level of *VgR* serves as a key molecular marker for assessing the ovarian developmental stage in *P. clarkii*. Its downregulation is directly associated with oocyte maturation and reproductive readiness in this species.

The *DMC* (disrupted meiotic cDNA) gene, a member of the RecA/Rad51 family, is recognized as a meiosis-specific gene in yeast [40]. Using RT-qPCR, Wang et al. [41] investigated the expression of the *DMC* gene across various tissues of the *P. clarkii*. The results demonstrated that the *DMC* gene is expressed in multiple tissues, including the gonads and hepatopancreas, with the highest expression levels observed in the testes and ovaries. Notably, the expression level in the testes was significantly higher than that in the ovaries, a finding consistent with the experimental results of Okutsu et al. [42].

Key transcription factors governing sex differentiation include the forkhead box protein L2 (*FOXL2*), crucial for ovarian development, and Doublesex- and Mab-3-Related Transcription factor 1 (*Dmrt1*) and SRY-Box Transcription Factor 9 (*Sox9*), predominantly associated with testicular development and spermatogenesis. Their expression is pivotal during gonadal differentiation and is modulated by both intrinsic signaling and external cues. Researchers have observed that the relative expression of the *Dmrt1* gene increases during the early stages of gonadal development in *P. clarkii*, suggesting that *Dmrt1* may participate in early sex differentiation and tissue morphogenesis in this species [43]. Similarly, the *Sox9* gene shows elevated expression during the early ovarian phase and the spermatogenic stage of *Scylla paramamosain*, indicating that it is a pivotal driver of gonadal differentiation and maturation [44]. *FOXL2* is a transcription factor that plays a pivotal role in animal sex differentiation and gonadal development [45]. The expression of *FOXL2* has been detected in the gonads of crustaceans such as *S. paramamosain* [45] and *P. clarkii*. Huang et al. [46] investigated the expression of *FOXL2* in *P. clarkii* and determined that its abundance in the testes significantly exceeded that in the ovaries. The expression level of *FOXL2* increased with the progression of gonadal development, peaking after gonadal differentiation, and played a crucial role in both testes development and early oocyte maturation in *P. clarkii*.

Studies have revealed that *cyclin B* and *cell division cycle 2* (*CDC2*) genes, as core components of the maturation-promoting factor (MPF), play a pivotal role in regulating oocyte meiosis and gonadal development in *P. clarkii*. Their expression exhibits a distinct stage-specific pattern: elevated during meiotically active phases (stages II and V) and suppressed during vitellogenic (stages III and IV), suggesting that these genes may act as key regulators during oocyte development [47,48].

Additionally, genes such as the proliferating cell nuclear antigen (*Pcna*) have been implicated in influencing gonadal development in shrimp [49]. Given the direct impact of gonadal development on the growth and reproduction of shrimp, further research on these core regulatory genes and the endogenous/exogenous factors controlling them remains essential.

## 3. Factors Influencing Gonadal Development in *Procambarus clarkii*

### 3.1. Environmental Factors

The external environment is a critical factor influencing gonadal development. Previous studies have demonstrated that environmental factors such as water temperature, light, salinity, and feed play significant roles in the gonadal development and sexual maturation of crustaceans [50,51,52]. Within a certain range, environmental cues interact with the neuroendocrine system and endogenous hormones, synergistically regulating gonadal development and maturation in crustaceans such as *P. clarkii*. However, exceeding this range may induce stress responses [53], thereby inhibiting gonadal growth and development.

#### 3.1.1. Temperature

As a poikilothermic species, *P. clarkii* is highly sensitive to temperature, which plays a pivotal role in its growth and development [54]. Research indicates that the optimal temperature range for *P. clarkii* growth is 20–32 °C, within which survival rates are higher, and increases in body length and weight are more pronounced [55,56]. Gonadal development in *P. clarkii* is also significantly influenced by temperature. Wang et al. [57] investigated the effects of elevated water temperatures on reproduction and found that within a specific range (18–26 °C), reproductive capacity and gonadal development were positively correlated with water temperature. Similarly, Xu et al. [58] observed that gonadal development accelerated with higher temperatures within the range of 22–28 °C, a finding consistent with Carmona-Osalde et al. [59], who reported higher gonadosomatic indices at elevated temperatures (16 °C, 21 °C and 26 °C). Analogous results were noted for *Procambarus llamasi*, where ovarian development rates increased with temperature within the range of 16–25 °C [59]. Furthermore, researchers have innovatively elucidated the specific protective mechanism of crustacean vitellogenesis under high-temperature conditions. Their findings demonstrate that at elevated temperatures (33 °C), certain crustaceans upregulate vitellogenin receptor (*VtgR*) gene expression through the VtgR enhancer–HSF1 pathway, thereby ensuring normal vitellogenin absorption and gonadal development [36]. In addition, excessively low temperatures (<18 °C) also delay gonadal development and significantly prolong egg incubation times [57]. These findings collectively demonstrate that water temperature regulates gonadal development in crustaceans via neuroendocrine pathways, which modulate the expression of key reproductive hormones and signaling pathways. Under suboptimal temperatures, impaired vitellogenin uptake by oocytes in *P. clarkii* disrupts gonadal maturation. Consequently, temperatures outside the optimal range (20–28 °C) impede normal gonadal development, whereas conditions within this range promote a positive correlation between thermal elevation and developmental progression.

#### 3.1.2. Salinity

Salinity is a critical ecological factor that plays a decisive role in the osmoregulation of aquatic organisms. It significantly influences the growth and gonadal development of crustaceans. When salinity fluctuations exceed their tolerance range, the internal homeostasis of these organisms is disrupted, leading to suppressed metabolic activity and, in severe cases, mortality [60,61]. Zhao et al. [62] demonstrated that at low salinity levels (<6 ppt), *P. clarkii* exhibited enhanced metabolic enzyme activity and upregulated heat shock protein (HSP90) expression. In contrast, high salinity (≥12 ppt) significantly suppressed metabolic activity and induced irreversible structural damage to the hepatopancreas and gill tissues. This physiological impairment indirectly compromises the energy allocation for reproduction and gonadal development, leading to a reduced reproductive capacity. Similarly, Meineri et al. [63] observed a significant decline in juvenile proportions at salinities > 5 ppt, with reproductive activities exclusively confined to water bodies below 5 ppt. This indicates that elevated salinity inhibits population-level reproductive success by compromising juvenile survival. Notably, studies have documented the presence of *P. clarkii* in saline environments. Nota et al. [64] reported its occurrence in high-salinity marine waters of the Mediterranean, highlighting its adaptability through metabolic regulation. However, survival does not equate to successful reproduction: Scalici et al. [65] observed in brackish wetlands of central Italy that this population exhibited reduced growth rates and a near absence of juveniles, indicating that high salinity inhibits its reproductive activities, especially mating, embryo development, and juvenile recruitment. In summary, prolonged exposure to non-optimal salinity adversely affects the immunity, metabolism, and reproductive capacity in *P. clarkii*, thereby impairing gonadal development directly and indirectly. Conversely, maintaining salinity within optimal ranges (juveniles < 5 ppt; adults < 6 ppt) enhances weight gain, growth rates, and gonadal development in *P. clarkii*.

#### 3.1.3. Light

*P. clarkii* is a benthic organism that prefers to inhabit areas with shelter, such as aquatic vegetation and branches, avoiding strong light and exhibiting nocturnal activity. Consequently, both light intensity and photoperiod serve as multifaceted ecological factors with diverse effects, directly or indirectly influencing the gonadal development of crustaceans [66]. Wang et al. [52] demonstrated that increasing the photoperiod significantly enhances the gonadosomatic index compared to complete darkness, a finding corroborated by transcriptomic analysis [67]. However, Su et al. [68] experimentally observed that excessive light exposure inhibits gonadal development in *P. clarkii*, highlighting the importance of an optimal and moderate photoperiod as a critical factor in promoting reproductive maturation. Light quality and wavelength also play a role in gonadal development. Studies have shown that natural light at 590 nm significantly improves the growth rates, whereas blue light (475 nm) and green light (525 nm) partially suppress reproductive development [69,70]. The optimal light intensity and photoperiod for gonadal development varies among decapod crustaceans (Table 6). In *Penaeus merguiensis*, high light intensity (1100 lx) induces regression of stage III–IV ovaries, whereas dim light (2 lx) significantly enhances the spawning rate and reproductive output [71]. Conversely, *Panulirus homarus* requires strong light stimulation (3350 lx) to promote gonadal maturation [72]. For *P. clarkii*, the ideal light cycle is 16 L:8 D (16 h of light and 8 h of darkness), with an optimal light intensity of 50–500 lx. Excessively high light intensity (>500 lx) disrupts normal physiological rhythms, impairs reproductive capacity, and hinders gonadal development [58]. This interspecific divergence is likely attributed to differential sensitivities of the X-organ–sinus gland (XO–SG) complex to light signals, which governs species-specific optimal light conditions for gonadal development [73]. Collectively, light intensity and photoperiod appear to act synergistically, coordinating endocrine pathways that drive gonadal development in crustaceans.

#### 3.1.4. Nutritional Conditions

The ovarian development of *P. clarkii* is a process of vitellogenesis, which serves as a critical prerequisite for oocyte maturation [77]. This process necessitates the continuous uptake of nutrients such as proteins and lipids, thereby establishing a close relationship between the nutritional intake, feed quality, and gonadal development in *P. clarkii* [78]. A growing body of research indicates that in the regulation of gonadal development, feed nutrition plays a more decisive role than environmental factors such as light and temperature [58,79].

Research indicates that higher levels of protein and fat in feed enhance the gonadal development and reproductive capacity in *P. clarkii* [80]. Jin et al. [81] demonstrated that the optimal dietary protein level for gonadal development in *P. clarkii* is 26%, which significantly enhances the muscle crude protein content while maintaining normal growth performance. Guo et al. [82] found that supplementing feed with 0.4% cholesterol significantly increased the gonadosomatic index and upregulated the expression of *vtg* and *vgr* genes in *Eriocheir sinensis*, thereby promoting vitellogenin accumulation. In *P. clarkii*, similar lipid-mediated regulation of ovarian development has been observed, where an optimal dietary cholesterol (0.5%) improved the antioxidant capacity and non-specific immune response, critical for vitellogenesis [83]. Furthermore, *P. clarkii* exhibits significant seasonal fluctuations in total lipids, triglycerides, and total caloric content, characterized by spring accumulation (peaking in April–June) and summer depletion (June–October). This dynamic is directly linked to gonadal development and vitellogenesis; however, it is independent of nutritional cycle regulation (evidenced by the absence of winter reserves). Consequently, triglycerides function as the primary energetic currency for reproduction-associated metabolism [27].

Beyond macronutrients, specific micronutrients are also vital for optimal gonadal development; the inclusion of appropriate vitamins (the ascorbic acid requirement of *P. clarkii* was estimated to be 265.67 mg/kg diet), trace elements (the selenium requirement: 10.02 mg/kg diet), and minerals (the phosphorus requirement: 13,900–14,300 mg/kg diet) in feed may also play an crucial role in enhancing gonadal development [84,85,86].

The influence of nutrients on ovarian maturation extends beyond their roles as substrates and signaling molecules; they may also exert effects through the modulation of neuroendocrine pathways. For instance, Se enhances gonadal development and spermatogenesis by regulating the expression of genes involved in sex hormone synthesis (such as testosterone and estradiol), including *Dmrt1* [86]. Thus, providing *P. clarkii* with optimal nutrition—characterized by sufficient high-quality protein, lipids, cholesterol, and specific levels of micronutrients—is crucial for influencing the extent of gonadal development and reproductive performance in this species.

#### 3.1.5. Toxic Substances

Heavy metals and pesticides, such as cadmium and copper, tend to accumulate in aquatic organisms such as crustaceans, posing significant risks to their growth and gonadal development when the concentrations exceed safe thresholds [87]. Yin et al. [88] demonstrated that hexavalent chromium (Cr (VI)) impairs ovarian development in *P. clarkii* by inducing oxidative stress (e.g., elevated malondialdehyde (MDA) levels), interfering with lipid metabolism (evidenced by upregulation of genes such as *PLA2XV-L*), and suppressing vitellogenin expression. Similarly, Yang et al. [89] found that cadmium disrupts ovarian development in *P. clarkii* by triggering oxidative stress in the hepatopancreas and inhibiting vitellogenin synthesis, which involves the downregulation of genes such as *hsp70* and *mih*. This aligns with findings reported by Yang et al. [90] in *Sinopotamon henanense*. Furthermore, copper induces excessive reactive oxygen species (ROS) production, resulting in oxidative damage to gonadal tissues of *P. clarkii* [91].

Atrazine, a broad-spectrum pesticide, primarily contains acetamiprid, which disrupts the nervous system of insects, causing paralysis and death. It is widely used in modern agriculture. Álvarez et al. [92] exposed varunid crab (*Neohelice granulata*) to varying atrazine concentrations (0, 2.5, 5 and 15 mg/L) for 32 days and observed a marked decline in oocyte numbers and vitellin content in the ovaries as atrazine levels increased. While these concentrations substantially exceed typical environmental levels (μg/L range), the study utilized them under controlled laboratory conditions to elucidate dose–response relationships and potential toxic mechanisms, explicitly noting the non-environmental realism of the higher doses. Similarly, Silveyra et al. [93] demonstrated that atrazine (5 mg/L) suppresses ovarian development in *P. clarkii* by inhibiting vitellogenin expression in the ovary and hepatopancreas, as well as disrupting the regulation of sex steroid hormones (e.g., estradiol). Additionally, this herbicide may indirectly impair vitellogenesis through non-hormonal pathways, such as energy metabolism redistribution. Collectively, these studies indicate that most toxic substances impair ovarian development in *P. clarkii* by inducing oxidative stress and suppressing transcription of key reproductive proteins (e.g., *Vtg* and *vg*). Moreover, certain toxins disrupt the production of key reproductive hormones, thereby hindering vitellogenesis. Thus, minimizing prolonged exposure of *P. clarkii* to toxic substances helps mitigate their adverse effects on gonadal development.

### 3.2. Eyestalk Ablation

The eyestalk serves as the neuroendocrine regulatory center in *P. clarkii*, playing a crucial role in its growth and development. It secretes the gonad-inhibiting hormone (GIH), which suppresses vitellogenin synthesis in female crayfish, and promotes the release of gonad-stimulating hormones. Comparative studies between eyestalk-ablated crustaceans and normally growing individuals have demonstrated that eyestalk removal accelerates molting and growth while enhancing gonadal development [94,95]. Similar conclusions have been drawn from eyestalk ablation experiments conducted on various crustaceans, including *S. paramamosain*, *M. rosenbergii* and *P. clarkii* [96,97,98]. However, eyestalk ablation inflicts physiological damage, potentially leading to reduced spawning rates or even mortality in broodstock [99]. Studies have demonstrated that eyestalk ablation reduces the reproductive efficiency (manifested as abnormal ovarian development and a significant increase in molting frequency to 337.5%), while significantly elevating broodstock mortality (15% in bilateral and 7.5% in unilateral ablation groups) [100]. To identify alternative methods for efficient maturation induction, researchers have investigated the molecular mechanisms underlying eyestalk–gonad interactions. Chen et al. [101] proposed that Vitellogenesis-Inhibiting Hormone (VIH) in the eyestalk may regulate vitellogenin synthesis indirectly via secondary messenger pathways, such as cyclic adenosine monophosphate (cAMP) and cyclic guanosine monophosphate (cGMP), and the Mitogen-Activated Protein Kinase (MAPK) pathway, thereby influencing gonadal development. Uawisetwathana et al. [102] further elucidated its association with gonadotropin-releasing hormone signaling and the maturation-promoting factor, highlighting a complex regulatory network involving multiple genes, molecules, and signaling pathways.

### 3.3. Hormone

**Figure 2:**
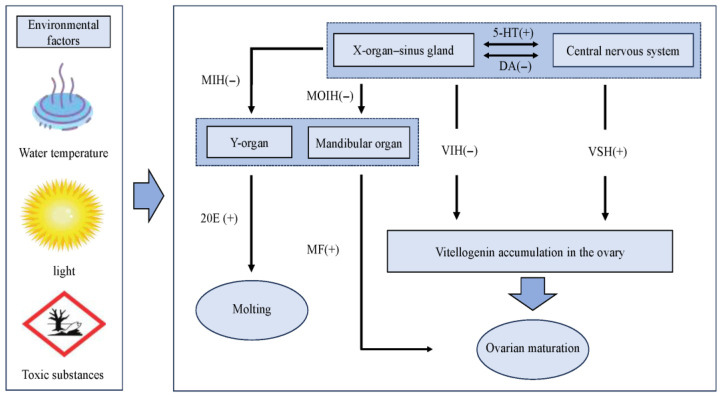
Schematic representation of hormonal regulation pathways governing molting and gonadal maturation in *P. clarkii*. 5-HT: 5-hydroxytryptamine, DA: dopamine, MIH: molt-inhibiting hormone, MOIH: maxillofacial organ-inhibiting hormone, 20E: 20-hydroxyecdy-sone, MF: methylfarnesylate, VIH: vitellogenesis-inhibiting hormone, VSH: vitellogenesis-stimulating hormone. (+) stands for promoting, (−) stands for inhibiting, and the double-line clippings represent bidirectional regulation.

Hormones are intrinsic factors influencing gonadal development in crustaceans [103,104]. In shrimp, the primary organs and tissues responsible for secreting hormones related to gonadal development include the X-organ–sinus gland (XO-SG), Y-organ, and mandibular organ [105]. As illustrated in Figure 2, these tissues and organs are intricately interconnected, collectively regulating shrimp growth and gonadal development. The XO-SG, located in the eyestalk of crustaceans, serves as a critical reproductive and endocrine regulatory system. It releases peptide hormones that modulate ovarian development. For instance, crustacean hyperglycemic hormone (CHH) significantly upregulates the expression of genes associated with gonadal development in *M. rosenbergii*, thereby promoting gonadal maturation [97]. Other notable hormones include GIH and molt-inhibiting hormone (MIH). The Y-organ, typically situated in the cephalothorax of crustaceans, primarily secretes ecdysteroids, which regulate molting behavior. Studies have revealed that ecdysteroid hormones exhibit elevated expression levels in the ovaries and embryos of certain shrimp and crab species during vitellogenesis [106,107]. Tiu et al. [108] revealed that 20-hydroxyecdysone (20 E), an active metabolite of molting hormones, significantly induces the upregulation of vitellogenin gene expression in *Penaeus monodon* hepatopancreatic tissue blocks cultured in vitro. The mandibular organ (MO), another essential endocrine gland in crustaceans, secretes methyl farnesoate (MF), an insect juvenile hormone analog. Studies have shown that MF directly stimulates vitellogenin expression and ovarian maturation [109]. Additionally, the MO secretes progesterone, which synergizes with MF to ensure normal ovarian growth and development [110].

The androgenic gland (AG) was first discovered in *Callinectes sapidus* and primarily secretes the insulin-like androgenic gland hormone (IAG). Studies have demonstrated its critical role in the development of male secondary sexual characteristics and spermatogenesis in crustaceans [111]. Yan et al. [112] observed that injecting IAG into *P. clarkii* significantly enhanced the digestive capacity of the hepatopancreas and accelerated gonadal development. In contrast, in the gonochoristic species *M.rosenbergii*, the expression levels of ovarian-derived IAG during ovarian development exhibited a negative correlation with vitellogenin gene expression [113]. These findings suggest that the regulatory role of IAG in gonadal development may differ among crustaceans.

Steroid hormones, small lipophilic molecules derived from cholesterol, are ubiquitously present in crustaceans [114]. Investigations into progesterone-mediated oocyte maturation pathways during ovarian development in *E.sinensis* revealed significant upregulation of progesterone signaling-related genes, suggesting its potential role in directly or indirectly promoting vitellogenesis and oocyte maturation [115]. Biogenic amines (BAs), widely distributed in crustacean peripheral organs and nervous systems, act as neurotransmitters to indirectly influence gonadal development through hormonal regulation [116]. Dopamine (DA), for instance, regulates immune responses and growth processes in crustaceans, thereby potentially influencing gonadal development indirectly via the neuroendocrine system [117,118], while serotonin (5-hydroxytryptamine [5-HT]) directly induces gonadal maturation [119].

## 4. Conclusions

**Figure 3:**
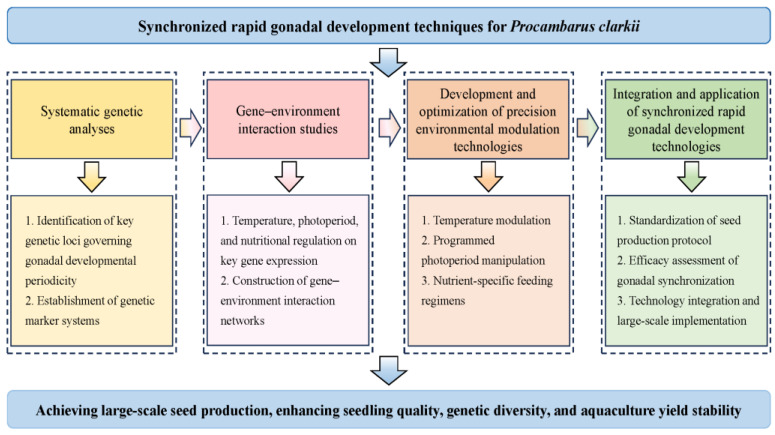
Roadmap for synchronized rapid gonadal development in *P. clarkii*.

Current research on gonadal development in *P. clarkii* has predominantly focused on histological structures, the developmental patterns of testes and ovaries, and oogenesis staging, while spermatogenesis remains understudied. Therefore, we propose the prioritization of investigations in the following areas. (1) Male gonadal development and influencing factors in *P. clarkii*: Current investigations predominantly focus on female specimens, with limited attention paid to male reproductive biology. Future research should prioritize detailed elucidation of the spermatogenic process (from spermatogonial proliferation through meiosis to spermiogenesis) in *P. clarkii*, coupled with the systematic identification of key environmental modulators (e.g., temperature, photoperiod, and endocrine signaling molecules) influencing male gonadal maturation. (2) Molecular mechanisms underlying gonadal regulation: Current research primarily addresses macroscopic aspects of gonadal development, while critical regulatory pathways remain elusive. Future studies should prioritize epigenetic regulation of key genes—such as *Dmc1* (driving meiotic recombination) and *VgR* (marking vitellogenesis)—through DNA methylation and histone modifications, which orchestrate gonadal maturation. Integrating transcriptomic and genomic approaches will unravel the detailed regulatory mechanisms and gene networks governing gonadal development. (3) Synchronized rapid gonadal development techniques for *P. clarkii*: Current juvenile production predominantly relies on in-pond self-propagation of parent stocks, a practice that inadvertently drives multigenerational inbreeding and counterproductive selection (e.g., size-selective harvesting), leading to genetic degradation. To mitigate this issue, it is essential to implement genetic management strategies, such as family-based selective breeding to maintain genetic diversity and genetic marker-assisted monitoring to counteract inbreeding depression. Asynchronous gonadal development results in heterogeneous juvenile sizes, elevated post-stocking mortality, and reduced yields. To address this, we propose integrating genetic management strategies with environmental modulation techniques to achieve synchronized rapid gonadal maturation in broodstock. First, systematic genetic analyses should identify the key loci governing developmental periodicity and establish corresponding genetic marker systems. Second, transcriptomic profiling must delineate the impact of environmental factors (e.g., temperature, photoperiod, and nutrition) on the expression of critical regulatory genes, thereby constructing gene–environment interaction networks. Furthermore, precision environmental control technologies—such as programmed photoperiod manipulation, temperature modulation, and nutrient-specific feeding regimens—require development and optimization to effectively synchronize gonadal development (Figure 3). Collectively, this integrated approach will establish a robust foundation for industrialized seed production, significantly enhancing seedling quality, genetic diversity, and aquaculture yield stability.

## Figures and Tables

**Table 1:** Histological characteristics of *P. clarkii* testes [9].

Structure	Morphological Characteristics	Function
Seminiferous tubules	Spherical units (10–16 μm) enveloped by connective tissue	Germ cell development
Collecting ducts	Three-tiered system: primary (connects single tubule), secondary (5–8 primaries converge), and tertiary (central lumen)	Sperm transport to vas deferens
Right vas deferens	Patent duct connection, secretory epithelium (anterior/middle), and thickened muscular wall (posterior)	Sperm transport and spermatophore formation
Left vas deferens	Junction sealed by epithelial filaments and no sperm content observed	Non-functional for sperm transfer

**Table 2:** Sperm developmental stages in *P. clarkii* [9].

Stage	Morphological Features of Spermatid Nuclei
Stage I	Nuclei appear capsule-shaped or sparsely pancake-shaped; nucleoplasm is sparse.
Stage II	Nuclei are nearly rounded; degree of nuclear condensation is higher than in Stage I.
Stage III	Nuclei are nearly rounded and slightly irregular; a distinct vesicular structure is apparent on one side of the nucleus.
Stage IV	Nuclei are smooth and rounded-quadrate; the vesicular structure is less conspicuous than in Stage III.
Stage V	Nuclei exhibit evident angular spines; a rounded structure is discernible within the nucleus.
Stage VI	Mature sperm exhibits a helical quadrangular shape; four distinct helically arranged radial arms extend from the nucleus; a distinct concentric structure is visible within the nucleus.

**Table 3:** Testes developmental stages and histological features in *P. clarkii* [15].

Stage	Month	Histological Features
Spermatogonia Stage	Jan–Mar	Seminiferous tubules are predominantly populated by spermatogonia and spermatocytes; mature sperm are seldom found in the vas deferens.
Sperm Maturation Stage I	Apr–Jun	The proportion of tubules containing spermatids and spermatozoa is markedly increased; sustentacular cells are prominent; the vas deferens is generally filled with mature sperm.
Spermatocyte Stage	Jul–Aug	Following sperm release, a new spermatogenic cycle commences; the proportion of tubules containing spermatocytes is significantly increased; spermatids at various metamorphic stages remain observable.
Sperm Maturation Stage II	Sep–Oct	The proportion of tubules containing spermatids and spermatozoa is significantly increased again (features analogous to Apr–Jun period); vas deferens is generally filled with mature sperm; empty tubules following sperm release are observable.
Spermatogonia Recovery Stage	Nov–Dec	Cells within the majority of seminiferous tubules revert to spermatogonia and spermatocyte stages (features analogous to Jan–Mar period); some tubules containing cells undergoing spermiogenesis are still discernible.

**Table 4:** The four phases of oogenesis in *P. clarkii* [20].

Phase	Features
Oogonia (proliferation phase)	The oocytes have large nuclei and little cytoplasm.
Pre-vitellogenic (small growth phase)	The oocytes increase rapidly in size, with an increase in cytoplasm, and the nucleoplasm is filled with granules.
Vitellogenic (large growth phase)	This phase is further divided into early, middle, and late stages. The oocytes continue to grow, and yolk granules start to accumulate and gradually increase in size.
Mature oocytes	At this stage, the nucleus disappears, the yolk granules are numerous and large in size, and the oocytes separate from the follicular cells.

**Table 5:** Morphological characteristics and staging of ovaries of *P. clarkii*.

Ovarian Stage	Color	Ovarian Morphology	References
Stage I	Grayish-white	Translucent thread-like structure, with oocytes indistinct to the naked eye, positioned medially to the hepatopancreas	[33]
Stage II	Pale yellow	Filamentous structure containing minute oocytes visible macroscopically; oocytes exhibit blurred boundaries and remain inseparable	[34]
Stage III	Yellow	Narrow ribbon-like structure with clearly outlined but incompletely developed oocytes	[35]
Stage IV	Light brown	Slender striated structure with discernible oocytes still adherent; follicular membranes become separable upon rupture	[29]
Stage V	Brownish-black	Rod-shaped structure containing uniformly plump oocytes that are minimally separable, fully occupying the cephalothoracic cavity	[15]
Stage VI	Dark brown	Fragile rod-shaped structure retaining only residual immature oocytes, with ovarian membranes prone to rupture	[30]

**Table 6:** Optimal light intensity and photoperiod for gonadal development in certain shrimp species.

Decapod Crustaceans	Types of Light Sources	Light Intensity	Photoperiod	Additional Parameters	References
*Penaeus merguiensis*	Daylight fluorescent tubes (36 W)	2 lx	12 L∶12 D	27 °C	[71]
*Panulirus japonicus*	Incandescent lamps (40 W)	70–100 lx	14 L∶10 D	25 °C	[74]
*Procambarus clarkii*	Fluorescent lamps (15–25 W)	50–500 lx	16 L:8 D	25–28 °C	[58]
*Lysmata vittata*	Tubular fluorescent lamps (25 W)	1000 lx	12 L∶12 D	25 °C	[73]
*Exopalaemon carinicauda*	LED retrofit tubes (5 W)	1000 lx	8 L:16 D	23–25 °C	[75]
*Cherax quadricarinatus*	Incandescent lamps (200 W)	3000 lx	16 L:8 D	28 °C	[76]
*Panulirus homarus*	Energy-saving lamps (80 W)	3350 lx	14 L∶10 D	29–32 °C	[72]

L: Light phase, D: Dark phase.

## Data Availability

No new data were created or analyzed in this study. Data sharing is not applicable to this article.
